# Alpha-Galacto-Oligosaccharides at Low Dose Improve Liver Steatosis in a High-Fat Diet Mouse Model

**DOI:** 10.3390/molecules22101725

**Published:** 2017-10-14

**Authors:** Eric Chappuis, Fanny Morel-Depeisse, Bruno Bariohay, Julien Roux

**Affiliations:** 1 Olygose, Rue les Rives de l’Oise, Venette 60200, France; fan.morel@gmail.com; 2 Biomeostasis, ActiParc I - Bat.6 Traverse de la Penne, La Penne-sur-Huveaune 13821, France; bruno.bariohay@biomeostasis.com (B.B.); julien.roux@biomeostasis.com (J.R.)

**Keywords:** NAFLD, pre-diabetes, metabolic syndrome, alpha-galacto-oligosaccharides, prebiotic

## Abstract

Non-Alcoholic Fatty Liver Disease (NAFLD) is the major liver disease worldwide and is linked to the development of metabolic syndrome and obesity. As alpha-galacto-oligosaccharides (α-GOS) from legumes have been shown to reduce body weight and hyperphagia in overweight adults, it was hypothesized that they would exert benefits on the development of metabolic syndrome and associated NAFLD in a rodent model. C57Bl/6J mice were fed a high-fat diet until they developed metabolic syndrome and were then orally treated either with α-GOS at a physiological dose (2.2 g/kg BW/d) or the vehicle over 7 weeks. α-GOS induced a reduction in food intake, but without affecting body weight during the first week of treatment, when compared to the vehicle. Fasting glycaemia was improved after 4 weeks of treatment with α-GOS, whereas insulin sensitivity (assessed with HOMA-IR) was unaffected at the end of the experiment. Plasma non-esterified fatty acids, low-density lipoprotein (LDL) and total cholesterol were lowered by α-GOS while high-density lipoprotein (HDL) and triglycerides levels remained unaffected. α-GOS markedly improved liver steatosis as well as free fatty acid and triglyceride accumulation in the liver. α-GOS improved plasma lipids and prevented NAFLD development through mechanisms which are independent of body weight management and glycemic control.

## 1. Introduction

Recent data suggest that the prevalence of metabolic disorders (such as obesity and diabetes) and liver diseases (such as non-alcoholic fatty liver disease, NAFLD) is growing. Between 1980 and 2013, the worldwide prevalence of overweight and obese adults (body mass index of 25 kg/m^2^ or greater) has increased from 28.8% to 36.9% in men and from 29.8% to 38.0% in women [1] while that of type 2 diabetes has increased from 4.3% to 9.0% in men and from 5.0% to 7.9% in women [2]. Obesity and diabetes development are commonly associated to NAFLD development which has become the most prevalent liver disease worldwide with figures ranging from 6% to 33% of the general population with a median prevalence of 20% [3,4,5]. NAFLD and type 2 diabetes share common physiopathological features, such as insulin resistance, both at a systemic and hepatic level [6] while obesity increases oxidative stress and free fatty acids (FFA) accumulation in the liver [7]. NAFLD can result in non-alcoholic steatohepatitis (NASH) and ultimately lead to cirrhosis and hepatocellular carcinoma [4] which are major mortality causes.

NAFLD management is currently based on body weight reduction through modifications of diet and physical activity [8]. Beyond classical lifestyle measures, few nutritional and pharmaceutical solutions have been proven efficient for the prevention and management of NAFLD, despite growing interest from the scientific community. Several solutions modulating cardiometabolic outcomes such as insulin sensitivity, weight loss, or dyslipidemia have also been tested in the prevention and treatment of NAFLD.

As the gut–liver axis is suspected to play a predominant role in the appearance of NAFLD [9], prebiotic oligosaccharides have gained interest and several have been tested for their effect on liver steatosis since the early 2000s. Inulin-type fructans have shown their ability to improve blood lipids and steatosis in various rodent models fed with high-fat or high-sugar diets [10,11,12]. Several modes of action have been proposed to explain the potential interest of prebiotic compounds in the prevention and management of NAFLD, including the improvement of plasma glucose and lipid control as well as direct effects on weight management [13,14,15] which can benefit patients.

Recent evidence suggests that alpha-galacto-oligosaccharides (α-GOS) extracted from legumes are new prebiotic compounds that can dose-dependently modulate appetite and weight gain in overweight adults [16]. Furthermore α-GOS have been proposed as possible solutions to regulate glucose metabolism and their effect has been recognized by European regulatory bodies through the acceptance of a health claim for food products [17]. Therefore, in this study we evaluated whether the effects of α-GOS on satiety, weight management, and glucose control may prevent, at a physiological dose (derived from Morel and al [16] and calculated according to the US food and drug administration recommendations [18]), the development of liver steatosis in mice fed a high-fat diet (HFD) for fifteen weeks, an animal model mimicking metabolic syndrome and NAFLD development [19].

## 2. Results

### 2.1. Development of Metabolic Syndrome in Animals

A total of 56 mice under HFD during the induction phase were considered as presenting a metabolic syndrome at the end of the induction phase and included in the treatment phase based on body weight (BW) and fasting glycaemia (FG).

### 2.2. Effect of α-GOS on Anthropometry and Food Intake

**Figure 1:**
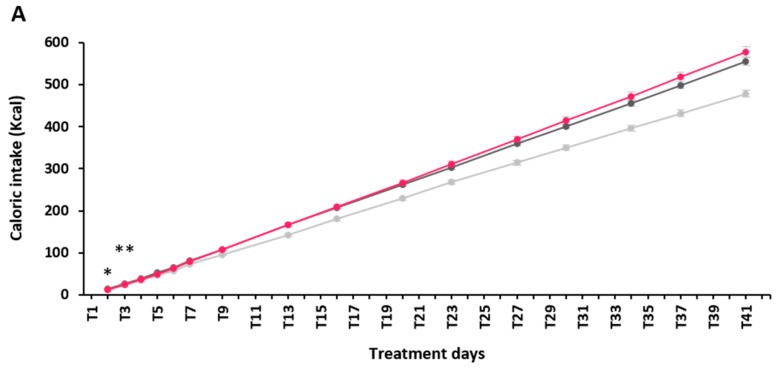

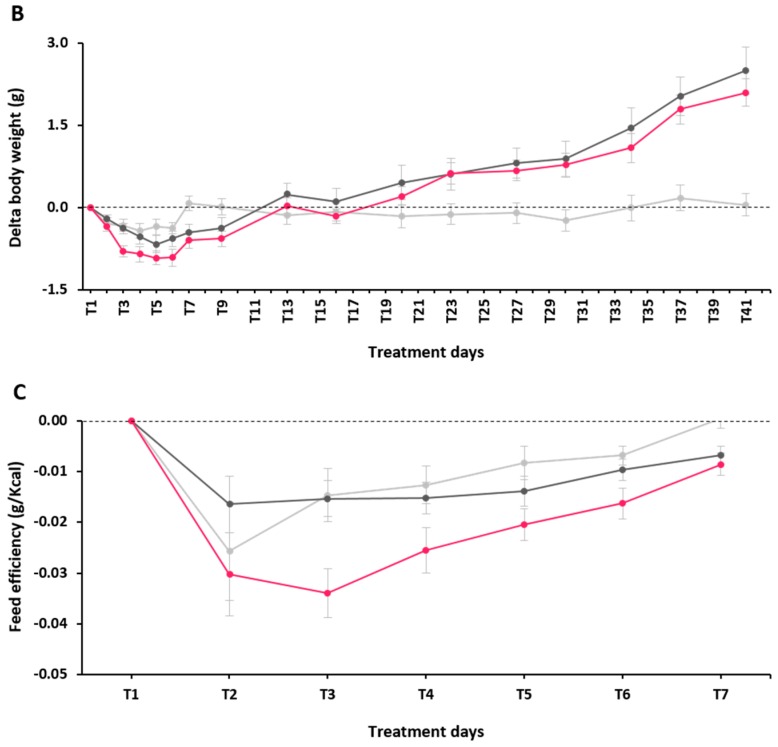
Effect of alpha-galacto-oligosaccharides (α-GOS) on anthropometric parameters and food intake during the treatment phase. (**A**) Caloric intake. (**B**) Daily delta body weight. (**C**) Feed efficiency during the first week of treatment phase. Data are mean ± standard error of the mean (SEM). Normal chow (NC), light gray lines, *n* = 16; high-fat diet (HFD)-control, dark grey lines, *n* = 20; HFD-α-GOS, pink lines, *n* = 20. * *p* < 0.05 vs. HFD-control group; ** *p* < 0.001 vs. HFD-control group; only statistical comparisons between HFD groups are shown. Treatment days (T) refer to the number of days after initiation of treatment with either the vehicle (NC and HFD-control groups) or α-GOS (HFD-α-GOS group).

Cumulative caloric intake (Figure 1A) was similar during the treatment phase among HFD-control and HFD-α-GOS groups, except at T2 and T3 (*p* = 0.029 and *p* = 0.007, respectively) where α-GOS-treated mice had lower caloric intake and at T4 and T5 where this difference approached significance (T4: *p* = 0.051; T5: *p* = 0.061). Daily body weight gain (Figure 1B) was not different between HFD groups at any time point during the treatment phase. No difference was observed except for total BW gain throughout the treatment phase (data not shown). Feed efficiency (Figure 1C) was not different among HFD groups during the first week, although close to significance (*p* = 0.070), which should rely on a clear downward trend for α-GOS treated mice, when compared to the control.

### 2.3. Effect of α-GOS on Plasma Parameters

**Figure 2:**
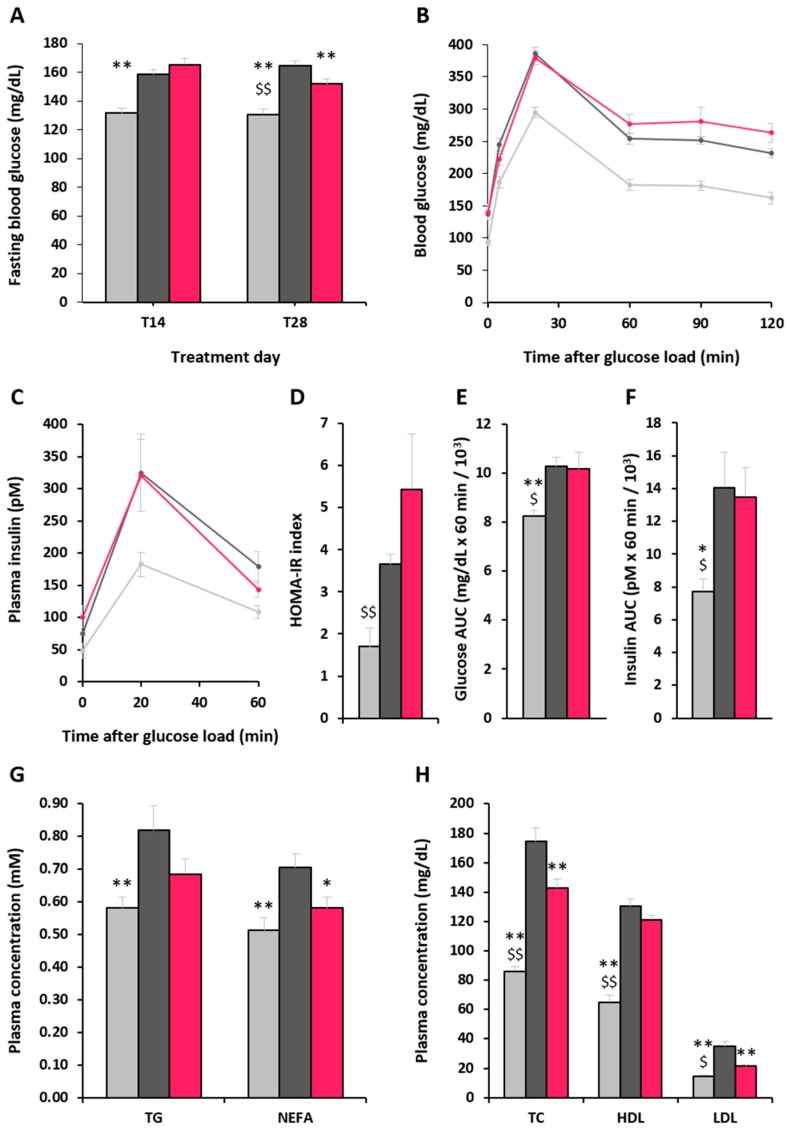
Effect of α-GOS on plasma parameters during the treatment phase. (**A**) Fasting glycaemia. (**B**) Glycemia kinetics after test meal (**C**) Insulinemia kinetics after test meal (D) HOMA-IR (**E**) Glycemia AUC after test meal (**F**) Insulinemia AUC after test meal (**G**) Triglycerides and non-esterified fatty acids. (**H**) Total cholesterol, HDL and LDL. Data are mean ± standard error of the mean (SEM). NC, light gray bars, *n* = 8 except for FG *n* = 16; HFD-control, dark grey bars, *n* = 10 except for FG *n* = 20; HFD-α-GOS, pink bars, *n* = 10 except for FG *n* = 20. * *p* < 0.05 and ** *p* < 0.001 vs. HFD-control group; ^$^ *p* < 0.05 and ^$$^ *p* < 0.001 for NC vs. HFD-α-GOS. Treatment days (T) refer to the numbers of days after initiation of treatment with either the vehicle (NC and HFD-control groups) or α-GOS (HFD-α-GOS group).

No difference between HFD groups was observed for FG 14 days after the initiation of treatment with either the vehicle or α-GOS (T14; *p* = 0.184), while at 28 days after initiation of treatment (T28), the HFD-α-GOS group showed a lower FG than the HFD-control group (*p* = 0.007) but still remained higher than the normal chow diet (NC) group (Figure 2A). The kinetics of plasma glucose (Figure 2B) and plasma insulin response (Figure 2C) after the meal challenge was not different in the HFD-control and HFD-α-GOS groups. Consequently, no difference was observed between these groups in the homeostasis model assessment of insulin resistance (HOMA-IR) index (Figure 2D) and in areas under the curve for plasma glucose and insulin (Figure 2E,F respectively). The NC group showed lower plasma glucose and insulin area under the curve (AUC) values when compared to both HFD groups, while HOMA-IR value was lower in the NC group compared to the HFD-α-GOS group (*p* = 0.005), but not different to the HFD-control group (*p* = 0.101). Plasma triglycerides (TG) levels (Figure 2C) were markedly increased in the HFD-control group when compared to the NC group (*p* = 0.007), while there was no difference between the HFD-α-GOS group and the NC group, as well as with the HFD-control group, despite a downward trend (*p* = 0.098). Plasma non-esterified fatty acid (NEFA) levels (Figure 2G) were significantly increased in the HFD-control group when compared to the NC group (*p* = 0.002), while NEFA levels of the HFD-α-GOS group were significantly lower than the HFD-control group (*p* = 0.028) and similar to those observed in the NC group. Total cholesterol (TC), HDL and LDL levels were markedly increased in the HFD groups when compared to the NC group. Nevertheless, TC and LDL levels were significantly decreased by α-GOS when compared to the HFD-control group (*p* = 0.003 and *p* < 0.001, respectively), but remained higher than in the NC group (Figure 2H). No difference was observed between HFD groups in HDL levels, which remained higher than those from the NC group (*p* < 0.001).

### 2.4. Effect of α-GOS on Liver Parameters

**Figure 3:**
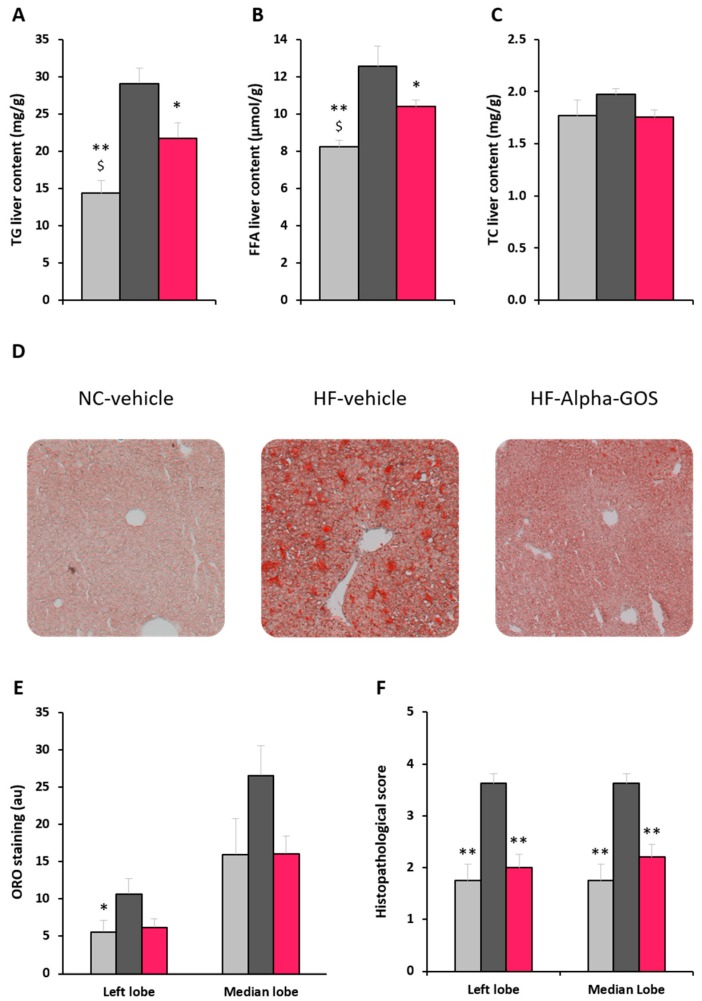
Effect of α-GOS on liver parameters during the treatment phase. (**A**) Hepatic triglycerides content. (**B**) Hepatic free fatty acids content. (**C**) Hepatic total cholesterol content. (**D**) Representative photographs of Oil-Red-O staining of the left liver lobe. (**E**) Oil-Red-O staining values (**F**) Histopathological scores for Oil-Red-O staining of liver sections. Data are mean ± standard error of the mean (SEM). NC, light gray bars, *n* = 8; HFD-control, dark grey bars, *n* = 10; HFD-α-GOS, pink bars, *n* = 10. * *p* < 0.05 and ** *p* < 0.001 vs. HFD-control group; ^$^ *p* < 0.05 for NC vs. HFD-α-GOS.

Hepatic TG and FFA levels were markedly increased in the HFD-control group when compared to the NC group (*p* < 0.0001 and *p* = 0.0003, respectively), while they appeared significantly decreased by the α-GOS treatment in comparison to the HFD-control group (*p* = 0.014 and *p* = 0.030, respectively), but remained significantly higher than those observed for the NC group (Figure 3A,B). TC liver contents were similar among all groups (Figure 3C). Oil-Red-O (ORO) staining differences approached significance (*p* = 0.068), but did not allow inter-group comparisons, despite clear differences in visual observation (Figure 3D,E). The left and median liver lobes’ histopathological scores were significantly increased in the HFD-control group when compared to the NC group (*p* < 0.0001 for both), while they were markedly decreased by α-GOS treatment when compared to control (*p* = 0.0002 and *p* = 0.0006, respectively), reaching a normalization when considering the absence of difference with the NC group (Figure 3F).

## 3. Discussion

The results of our experiment suggest that α-GOS exerts beneficial metabolic effects in the context of a high-fat diet, more particularly on lipid profile and liver steatosis. These results are of primary interest as the dose of α-GOS used in this study is by far lower than the doses of prebiotic fibers used in other similar experiments with rodents. Generally, prebiotics have been shown to exert similar effects in rodents at levels around 5–10% of food intake (in weight) [11,12] while in our experiment we tested a more physiological dose of 1% of food intake (in weight). This is a major consideration when generating new insights into the effects of nutritional compounds on health as the ability to transpose findings from animals to humans remains a huge challenge for the scientific community.

The low dose used in this study may explain a number of findings. Treatment with α-GOS resulted in limited improvements in anthropometric variables throughout the experiment. We observed a transient anorectic effect of α-GOS during the first week of treatment, and interestingly, this anorectic effect did not affect body weight gain and was not explained by a change in feed efficiency. These results echo only partially those observed in humans at equivalent doses where α-GOS treatment improved body weight after 2 weeks and improved satiety during a test meal in overweight adults [16]. The limited transposition of the results from humans to animals in this study might be explained on one hand by the type of diet used to induce metabolic syndrome (high-fat diet), an occurrence that is highly unlikely in humans, and on the other hand, by the fact that only male mice were included in the protocol to avoid any disturbance impact from hormonal cycle on eating behavior. Nevertheless, most oligosaccharides administered to rodents under a high-fat diet have been found to be effective on body weight and adiposity, but only when fed at higher levels, around 10% of food intake (by weight) [20,21]. The impact of prebiotic oligosaccharides on appetite and satiety hormones has been investigated in a number of studies, but resulted in inconsistent results in humans. To our knowledge, limited data are available on the effects of dietary fibers (and more particularly prebiotics) on the secretion of cholecystokinin (CCK) in humans [22]. ON the contrary more studies have investigated the impact of prebiotic supplementation on GLP-1 secretion in rodents fed high-fat diets and in humans, but these studies have led to erratic results. While oligofructose seems to positively affect GLP-1 in diabetic rats fed high-fat diets [23], results in humans with respect to chronic supplementation have been conflicting [24,25]. This is also the case with the modulation of glucose-dependent insulinotropic peptide (GIP) [25,26,27]. The measurement of peptide YY (PYY) and ghrelin could also have revealed possible mechanisms of action of α-GOS to improve hyperphagia during the first week of treatment [25,26,27]. These appetite and satiety mediators are partly regulated by the release of short-chain fatty acids (SCFAs) and other SCFA-independent mechanisms [22,28,29]. As several human trials have backed prebiotic oligosaccharides for their ability to modulate these hormones, additional analyses on these mediators at the end of the intervention could have revealed other possible mechanisms of action.

Some improvements of plasma parameters related to glycemic control, such as a decrease in fasting glycaemia after four (but not two) weeks of treatment, were observed throughout the experiment. This finding suggests that longer-term treatment would be required to observe a sustained effect and an additional measurement point at the end of the treatment period could have backed this hypothesis. This finding is interesting and contrasts with results from human interventional trials showing limited benefits of prebiotic oligosaccharides on glycemic control in overweight or obese adults, whatever their diabetic status [30].

The plasma lipid profile was improved by α-GOS treatment by decreasing NEFA, TC and LDL levels compared to the HFD-control group. A downward trend for TG was also observed. However, more importantly, α-GOS brought TG and NEFA levels back to normal values with no difference observed with the NC group. These results are consistent with existing literature showing that prebiotic oligosaccharides improve blood lipid parameters, namely TC and LDL, in overweight and obese patients [30], while the improvement of HDL and TG levels generally observed in diabetics has not been backed by our experiment, despite a downward trend for TG levels. Though several explanations have been suggested, the exact mechanisms by which prebiotic compounds exert an effect on lipid profile remain poorly understood. First, the effect of prebiotics on gut hormones such as GLP-1 leading to improved insulin resistance together with the improvement in post-prandial glycaemia have been proposed [31]. Second, the modulation of SCFAs secretion by the gut microbiota, especially a decrease in acetate (converted to acetyl-CoA where it acts as a substrate for fatty acid synthesis in hepatocytes), has been proposed [32], but lacks interventional supports as GOS have been proven both to increase acetate production and to decrease lipidemia [33,34]. Other mechanisms include enzymatic deconjugation of bile salts by bacteria, cholesterol binding in the small intestine, incorporation of lipids into bacterial cellular membranes during growth, conversion into coprostanol, and fecal excretion [35], but these parameters have not been assessed in our experiment.

The treatment with α-GOS markedly improved liver parameters in our experimental setting. Hepatic TG and FFA levels were decreased compared to the HFD-control group, while TC seemed to decrease, but high inter-individual variability did not allow us to reach statistical significance. Similarly, the ORO staining seemed to be improved with α-GOS, but variability in the results did not allow us to draw definitive conclusions on this parameter. Finally, the steatosis level measured by histopathological scoring revealed the ability of α-GOS treatment to normalize this feature. This effect was not mediated by weight loss and the subsequent normalization of metabolic status [13] as we primarily hypothesized. Current NAFLD management is based on lifestyle modifications that aim to reduce body weight [8] and can include hypolipidemic therapy when appropriate [36,37], suggesting that the improvement of the plasma lipid profile may be one of the factors responsible for the normalization of liver parameters with α-GOS treatment. Other effects can be envisaged as current evidence suggests that microbiota affects insulin resistance, fatty liver, fibrosis and the necroinflammatory score, which are all involved in the accumulation of triglycerides in the liver [38]. The possible mechanisms of action involved include modulation of FIAF (fasting-induced adipocyte factor), modulation of bile acids through Farnesoid X receptors (FXR), direct and indirect effects of SCFAs produced by the gut microbiota, or the modulation of low-grade inflammation through modulation of bacterial lipopolysaccharides (LPS) production and intestinal permeability linked to metabolic endotoxemia [9]. Interestingly, α-GOS has been shown to decrease metabolic endotoxemia by modulating plasma lipopolysaccharide (LPS) and ultra-sensible C-reactive protein (usCRP) levels in overweight adults [16], suggesting an improvement mediated by a decrease in intestinal permeability. However, our experimental design did not intend to measure these features.

The main limitation of the study lies in the absence of any measurement of microbiota-related effects, such as SCFAs, microbiota structure or metabolic endotoxemia, which could have provided additional insights into the benefits of α-GOS in NAFLD, because our initial hypothesis was that weight loss and glycemic normalization would lead to an improvement in NAFLD. Additional experiments aimed at evaluating the impact of microbiota-related improvements would provide further explanations on the possible mechanisms of the actions involved. Furthermore, it can be hypothesized that the study design of splitting animals into two groups for evaluation, either along glycemic parameters or liver parameters, has decreased the statistical power of these analyses. For example, in the case of ORO staining, performed with only 10 animals in each group, visual observation suggests a clear inter-group difference, while the ANOVA step only reached a trend to significance and did not allow for further statistical exploration. Finally, the liver histology assessment methodology used in this study did not include an assessment of inflammation or fibrosis, and relied only on hepatocellular lipid content. The choice of this assessment was made considering that the short intervention period with HFD generally does not allow for the observation of the apparition of such symptoms in mice, but prevents the use of validated histopathological scores, such as the Brunt score or SAF (steatosis, activity and fibrosis) score. Furthermore, additional information on liver functioning would have been gathered through the measurement of liver enzymes such as gamma glutamyltransferase or transaminases.

In conclusion, α-GOS improves plasma lipid parameters and prevents NAFLD development through weight and glycemic control-independent mechanisms in a mice model of NAFLD.

## 4. Materials and Methods

### 4.1. Animals and Experimental Design

The animal experiment was conducted at Biomeostasis (Marseille, France) and was approved by the French Ministry of Higher Education and Research and the Local Ethic Committee of Provence n°14 under project number 01532.02, in strict accordance with European Economic Community Guidelines (86/609/EEC). A total of 56 male C57Bl/6J mice at 5 weeks of age were purchased from Janvier Labs (Saint Berthevin, France). Mice were housed collectively in standard plastic cages (*n* = four-five/cage) and maintained in a temperature- (24.0 to 26.0 °C) and humidity- (40.0 to 50.0%) controlled room on a 12-h light (7:00 a.m.–7:00 p.m.)/12-h dark cycle. After one week adaptation with ad libitum access to water and standard pellet food (pellet AO4; SAFE, Villemoisson-sur-Orge, France), animals either continued standard pellet food (Normal Chow, NC) or were fed a high-fat diet (HFD; 60% of energy from fat, SSNIFF, Soest, Germany) for eight weeks (Induction phase), before starting the treatment phase. On the seventh week of the induction phase, mice fed HFD which developed a metabolic syndrome phenotype (compared to NC mice) on the basis of their body weight (BW) and fasting glycaemia (FG) levels were selected for the subsequent experimentations. Mice were then matched into one NC group and two HFD groups based on these parameters and daily habituated to oral gavage, once a day, for one week before the treatment phase.

Following this habituation period, the NC group and one HFD group were daily treated with the vehicle while the remaining HFD group was treated with α-GOS (α-GOS, CravingZ’Gone^®^, Olygose, Venette, France) at a dose of 2.2 g/kg BW/day during 6 to 7 weeks (treatment phase). During the treatment phase, BW and food intake (FI) were monitored daily for the first week and twice a week for the following weeks. Food intake and BW were measured using a precision scale (THB-600 G, PMC Millot; precision ± 0.01 g). Feed efficiency was calculated as follows: BW gain (g)/cumulative caloric intake (Kcal).

At the end of the second and fourth weeks of treatment, semi-fasted (3 h of fasting in the early morning) glycaemia was measured using an ACCU-CHEK^®^ Performa glucometer (Roche diagnostics, Meylan, France).

At the end of the sixth week, each group of mice was divided into two matched subgroups (based on FG and BW) in order to perform either an Oral Glucose Tolerance Test (OGTT) after an overnight fasting.

For OGTT, overnight-fasted mice (15 h) were given a glucose solution (1.5 g/kg body weight) by oral gavage. Blood samples were collected at times 0, 15, 30, 60, 120 and 240 min following glucose administration. Glycaemia was measured at all time points while insulinemia was measured at time points 0, 15 and 60 min after glucose administration. Mice performing the OGTT were allowed to recover for one additional week of treatment before being anesthetized (100 mg/kg ketamine and 10 mg/kg xylazine (Centravet, Dinan, France)) for liver and blood sampling. Left liver lobes were immediately snap-frozen in liquid nitrogen and stored at −80 °C for hepatic lipid analysis.

### 4.2. Biochemical and Histological Analyses

Plasma insulin concentrations were measured using an ultrasensitive insulin ELISA kit (ALPCO Diagnostics, Salem, NH, USA) after plasma centrifugation (2000× *g*, 15 min, 4 °C). The homeostasis model assessment of insulin resistance (HOMA-IR) was calculated according to the following formula: HOMA-IR = Fasting insulin (microU/L) × fasting glucose (mmol/L)/22.5.

Plasma triglycerides were analyzed using the EnzyChrom Triglyceride Assay kit (BioAssay Systems, Hayward, CA, USA). Plasma non-esterified fatty acids (NEFA) were measured using the Wako NEFA-HR(2) assay kit (Wako Chemicals, Richmond, VA, USA). Plasma high-density lipoprotein (HDL), low-density lipoprotein/very low-density lipoprotein (LDL/VLDL), and total cholesterol (TC) were determined using the EnzyChrom AF Cholesterol Assay kit (BioAssay Systems, Hayward, CA, USA). All experimental assays were performed according to the manufacturer’s instructions.

Total lipid extractions were performed according to the Folch method. Total lipids were extracted using ethanol:chloroform (1:2; vol/vol) and then heated in a water bath at 56 °C for 3 min. After addition of 0.58 mL of saline (0.9% NaCl) and centrifugation (1620× *g*, 10 min), organic phases were extracted and concentrated under a stream of argon gas and stored for biochemistry assays. Triglycerides and cholesterols were quantified by spectrophotometer (Spectro-fluorometer Tecan Infinite, Männedorf, Switzerland) using triolien and cholesterol as internal standards respectively. Fatty acids were quantified by fluorometer (Spectro-fluorimeter Tecan Infinite, Männedorf, Switzerland) using palmitic acid as an internal standard. All values were expressed per gram of liver sample.

For hepatic lipid extraction and analysis, samples (~200 mg) of frozen liver were crushed in tubes placed on cold ice. Total lipid extractions were performed according to the Folch method. Total lipids were extracted using ethanol:chloroform (1:2; vol/vol) and then heated in a water bath at 56 °C for 3 min. After addition of 0.58 mL of saline (0.9% NaCl) and centrifugation (1620× *g*, 10 min), organic phases were extracted and concentrated under a stream of argon gas and stored for biochemistry assays. Triglycerides and cholesterols were quantified by a spectrophotometer (Spectro-fluorometer Tecan InfiniteMännedorf, Switzerland) using triolien and cholesterol as internal standards respectively. Fatty acids were quantified by a fluorometer (Spectro-fluorimeter Tecan Infinite) using palmitic acid as an internal standard. All values were expressed per gram of liver sample.

For liver histologic evaluation, the samples were washed with PBS, post-fixed in 3% potassium dichromate solution and washed again in PBS. Samples were cut with a disposal blade under a stereo zoom microscope and each lobe was placed in a plastic mould containing cryo-compound (OCT) and snap frozen. The samples were sliced (7 µm thickness) with a cryostat (Leica CM3050, Nanterre, France) and collected on a SuperfrostPlus microscope slide. The slides were stained with Oil Red O (ORO) solution (0.5% in propylene glycol). Briefly, the slides were placed in propylene glycol. An experienced anatomopathologist blinded to the treatment groups evaluated the liver sections. For each animal, 2 frozen liver sections (median and left lobe) were assessed without knowledge of the treatment groups and regimen status, first as a primary read consisting of assigning scores on left and median lobes, with comments as appropriate. After all slides were primarily reviewed, hepatocellular lipid content was scored from 0 to 6 (0: no detection of lipid vacuoles; 5: very marked lipid content, i.e., full blown lipidosis/steatosis/fatty degeneration with coalescing areas of predominantly macrovesicular lipidosis and a predominance of hypertrophic hepatocytes mimicking morphologically adipocytes) after all slides were primarily reviewed. Finally, 2 images of each animal were analyzed with ImageJ software. The amount of lipids was assessed by the quantification of tissue lipid accumulation via the amount of ORO staining.

### 4.3. Statistical Analyses

All data are represented as mean ± SEM. Statistical analyses were performed with the Statview 5.0.1 program (Statview software, Cary, NC, USA) and GraphPad Prism. Data were analyzed by Student test, one-way ANOVA or repeated-measures ANOVA. A Student test was used to compare the HF group versus the NC group during the Induction Phase and applied for the analysis of the Oil Red O quantification between the four different experimental groups. Repeated-measures ANOVA was applied for the analysis of the body weight gain, the cumulative food intake and glycaemia during the Treatment Phase. On the other hand, simple ANOVA analysis was applied for comparisons of the mean value of a given parameter between the three different experimental groups. When an effect was significant, a post-hoc analysis was performed with a Fisher test PLSD. The risk α was fixed at 0.05.
